# Fatty acids in non-alcoholic steatohepatitis: Focus on pentadecanoic acid

**DOI:** 10.1371/journal.pone.0189965

**Published:** 2017-12-15

**Authors:** Wonbeak Yoo, Donjeta Gjuka, Heather L. Stevenson, Xiaoling Song, Hong Shen, Suk Young Yoo, Jing Wang, Michael Fallon, George N. Ioannou, Stephen A. Harrison, Laura Beretta

**Affiliations:** 1 Department of Molecular and Cellular Oncology, The University of Texas MD Anderson Cancer Center, Houston, Texas, United States of America; 2 Department of Pathology, University of Texas Medical Branch, Galveston, Texas, United States of America; 3 Division of Public Health Sciences, Fred Hutchinson Cancer Research Center, Seattle, Washington, United States of America; 4 Department of Bioinformatics and Computational Biology, The University of Texas MD Anderson Cancer Center, Houston, Texas, United States of America; 5 Division of Gastroenterology, The University of Texas Health Science Center at Houston, Houston, Texas, United States of America; 6 Division of Gastroenterology, Veterans Affairs Puget Sound Health Care System and University of Washington, Seattle, Washington, United States of America; 7 Department of Medicine, Brooke Army Medical Center, San Antonio, Texas, United States of America; Institute of Medical Research A Lanari-IDIM, University of Buenos Aires-National Council of Scientific and Technological Research (CONICET), ARGENTINA

## Abstract

Non-alcoholic fatty liver disease (NAFLD) is the most common form of liver disease and ranges from isolated steatosis to NASH. To determine whether circulating fatty acids could serve as diagnostic markers of NAFLD severity and whether specific fatty acids could contribute to the pathogenesis of NASH, we analyzed two independent NAFLD patient cohorts and used the methionine- and choline-deficient diet (MCD) NASH mouse model. We identified six fatty acids that could serve as non-invasive markers of NASH in patients with NAFLD. Serum levels of 15:0, 17:0 and 16:1n7t negatively correlated with NAFLD activity scores and hepatocyte ballooning scores, while 18:1n7c serum levels strongly correlated with fibrosis stage and liver inflammation. Serum levels of 15:0 and 17:0 also negatively correlated with fasting glucose and AST, while 16:1n7c and 18:1n7c levels positively correlated with AST and ferritin, respectively. Inclusion of demographic and clinical parameters improved the performance of the fatty acid panels in detecting NASH in NAFLD patients. The panel [15:0, 16:1n7t, 18:1n7c, 22:5n3, age, ferritin and APRI] predicted intermediate or advanced fibrosis in NAFLD patients, with 82% sensitivity at 90% specificity [AUROC = 0.92]. 15:0 and 18:1n7c were further selected for functional studies *in vivo*. Mice treated with 15:0-supplemented MCD diet showed reduced AST levels and hepatic infiltration of ceroid-laden macrophages compared to MCD-treated mice, suggesting that 15:0 deficiency contributes to liver injury in NASH. In contrast, 18:1n7c-supplemented MCD diet didn’t affect liver pathology. In conclusion, 15:0 may serve as a promising biomarker or therapeutic target in NASH, opening avenues for the integration of diagnosis and treatment.

## Introduction

Non-alcoholic fatty liver disease (NAFLD) is rapidly becoming the most common form of liver disease worldwide. NAFLD is defined as evidence of hepatic steatosis, either by biopsy or imaging, in the absence of substantial alcohol intake, viral hepatitis, steatogenic medications or congenital metabolic disorders.[1] NAFLD has been proposed as the hepatic manifestation of the metabolic syndrome.[2–4] NAFLD presents as a spectrum of histological states from isolated steatosis to hepatic inflammation known as non-alcoholic steatohepatitis (NASH). NASH may further progress to cirrhosis and hepatocellular carcinoma (HCC).[5] Patients with NASH are those at greatest risk of progression to cirrhosis and, thus, diagnosis and treatment efforts should be targeted to these individuals.[5, 6] Although the diagnosis of NAFLD is straight forward, a liver biopsy is currently required to diagnose NASH. However, liver biopsy has a number of limitations, including sampling error, cost, and risk of complications. Furthermore, it is not feasible to perform liver biopsies on all NAFLD patients.[7, 8]

Lipid accumulation is a central feature in NAFLD and results from multiple contributors ranging from increased fatty acid flux to *de novo* lipogenesis. Increased lipogenesis is a major characteristic of NAFLD and appears to be a more critical source for steatosis than diet.[9, 10] We recently reported that in a mouse model of NASH, animals fed a regular diet developed alterations in both hepatic and circulating fatty acid composition, in agreement with an important role for *de novo* lipogenesis in NASH.[11] Therefore, we aimed to determine whether circulating fatty acids could serve as diagnostic markers of NAFLD severity and whether specific fatty acids could contribute to the pathogenesis of NASH. To that end, we analyzed two independent NAFLD patient cohorts and used the methionine- and choline-deficient diet (MCD) NASH mouse model. A recent study provided evidence that feeding mice a MCD diet is a valid model of the pathobiological mechanisms that cause human NAFLD to progress to advanced NASH. [12]

## Materials and methods

### Patient cohorts

This study included 106 patients with non-alcoholic fatty liver disease (NAFLD) who underwent biopsy at Brooke Military Hospital in San Antonio (n = 75) and the Veterans Affairs Puget Sound Health Care System in Seattle (n = 31). Demographic and clinical information of patients are summarized in Table 1. Written, informed consent was obtained from every human subject when the samples were originally collected for the repositories under the terms of study protocols approved by the Institutional Review Boards at Brooke Military Hospital and the Veterans Affairs Puget Sound Health Care System. NAFLD was defined as the presence of hepatic steatosis in at least 5% of hepatocytes, in the absence of HCV RNA and hepatitis B virus surface antigen, in the context of less than 20g/day for women and 30g/day for men of alcohol consumption in the preceding six months, and absence of histological features suggestive of another etiology of liver disease. Isolated steatosis was defined as the presence of hepatic steatosis without inflammation, ballooning or fibrosis. Liver biopsy interpretation was performed locally by pathologists using the criteria reported by Brunt et al.[13] The stage of fibrosis was assessed using a zero to four scale (0 = no fibrosis; 1 = mild/moderate zone three perisinusoidal fibrosis, or portal/periportal fibrosis only; 2 = perisinusoidal and portal/periportal fibrosis; 3 = bridging fibrosis; 4 = cirrhosis). Scores for steatosis grade (0–3), ballooning (0–2) and lobular inflammation (0–3) were also recorded. NAFLD Activity Score (NAS) is the unweighted sum of steatosis, lobular inflammation, and ballooning scores.

**Table 1:** Characteristics of the patient population.

	NAS 1–4 (*n* = 68)	NAS ≥5 (*n* = 38)	*p*-value
**Age**	47 (26–71)	51 (25–64)	ns
**Gender (% male)**	75	66	ns
***Ethnicity***			ns
**White (%)**	55	62	
**Hispanic (%)**	28	17	
**Asian (%)**	13	10	
**African American (%)**	4	10	
**Total Bilirubin (mg/dl)**	0.50 (0.2–1.3)	0.55 (0.2–1.3)	ns
**ALT (IU/L)**	59 (14–170)	83 (21–183)	0.003
**AST (IU/L)**	33.5 (15–135)	54.5 (18–162)	<0.001
**Creatinine (mg/dl)**	0.9 (0.5–1.8)	0.9 (0.5–4.5)	ns
**Albumin (g/dl)**	4.4 (3.4–5)	4.4 (3.5–5.4)	ns
**LDL (mg/dl)**	113.5 (44–224)	104.0 (43–173)	ns
**HDL (mg/dl)**	41.0 (25–70)	40.5 (25–74)	ns
**Triglycerides (mg/dl)**	142 (51–1007)	183 (75–407)	ns
**Glucose (mg/dl)**	102 (79–302)	109 (77–257)	ns
**Insulin (mIU/ml)**	18.5 (0.3–134.9)	23.1 (4.8–282)	ns
**White blood cell count (1000/μl)**	6.7 (2.9–10.8)	7.4 (4.6–14.1)	ns
**Platelet count (1000/μl)**	240.5 (79–629)	234.0 (83–504)	ns
**Ferritin (ng/ml)**	212.3 (22–690)	312.0 (73–1858)	0.02
**Hemoglobin (g/dl)**	14.6 (12.4–16.9)	14.3 (9.2–17.5)	ns
**APRI**	0.4 (0.2–1.6)	0.8 (0.3–2.1)	<0.001

All data expressed as median (range) unless otherwise specified. ns: not significant. ALT, Alanine transaminase; AST, Aspartate transaminase; NAS, NAFLD activity score; LDL, low-density lipoprotein; HDL, high-density lipoprotein; APRI, (AST/ULN)/platelets(10^9^/L)x100 where ULN is the accepted upper limit normal level of AST (33 IU/L).

### Fatty acid profiling

Fatty acid profiling was performed on fully de-identified serum samples that were transferred from sample repositories. Total lipids were extracted from 100 μL of human serum as previously described.[14] The phospholipid fraction was isolated by one-dimensional thin-layer chromatography.[15] Methyl esters of phospholipid fatty acids were prepared using direct transesterification[16] and separated by gas chromatography (Agilent 7890 Gas Chromatograph with FID detector and ChemStation software; Supelco fused silica 100 m capillary column SP-2560; 160°C for 16 minutes, ramp 3°C/minute to 240°C and hold for 15 minutes). The assay generated data on 46 fatty acids, and the fatty acid composition is expressed as weight percentage of the total fatty acids analyzed in the phospholipid fraction.

### Mice treatment

The study was approved by theInstitutional Animal Care and Use Committee (IACUC) at MD Anderson Cancer Center. Eight week-old male C57BL/6 mice were randomly divided into three groups (n = 8–10 mice per group) as follows. Group 1: methionine- and choline-deficient diet (MCD); Group 2: MCD diet containing 5% pentadecanoic acid (Sigma Chemical, St. Louis, MO) (MCD+15:0) and Group 3: MCD diet containing 5% vaccenic acid (Nu-Check-Prep, Inc., Elysian, MN) (MCD+18:1n7c). All diets were prepared by Harlan Teklad (Madison, WI). The addition of 15:0 or 18:1n7c fatty acids replaced 5% of the fat content in the MCD diet so that all diets were comparable in total fat content. Mice were treated for four weeks. IACUC-endorsed euthanasia by CO_2_ was performed followed by necropsy at which time blood was obtained by cardiac puncture. Aspartate aminotransferase (AST) and alanine aminotransferase (ALT) serum levels were measured using ACE Axcel Clinical Chemistry Analyzer (Alfa Wassermann Diagnostic Techno-logies, LLC, West Caldwell, NJ). Liver tissue was also collected and fixed in 10% neutral buffered formalin for histology analysis and Masson’s trichrome, and the number of PASD-positive macrophages was counted. All counts were performed using the ×20 objective. Macrophages were included in the count only if they were clearly distinguishable from the surrounding cells and contained more PASD-positive material than the size of the nucleus. Staining quantification was performed using Aperio scanner (Aperio AT2) and software. Histological diagnosis was performed blindly by a pathologist and histopathologic lesions of the liver sections were scored using the criteria reported by Brunt et al.[13]

### Real-time quantitative PCR (qRT-PCR)

Total RNA was extracted from individual mouse liver tissues with the miRNeasy extraction kit (Qiagen). RNA samples were submitted to reverse transcription and real-time PCR. The following primers were used: IL-6 (F 5’- CGGAGGCTTAATTACACATGTTCTC-3’, R 5’- CAGTTTGGT AGCATCCATCATTTCT-3’) and TNF-ɑ (F 5’-ACGGCATGGATCTCAAAGA C-3’, R 5’- AGA TAGCAAATCGGCTGACG -3’). cDNA equivalent was then amplified with the CFX connect real time system using SYBR green supermix and analyzed by CFX manager software (Bio-Rad Laboratories) and relative quantification of RNA expression was calculated with the 2^-ΔΔCt^ method. GAPDH was used for normalization.

### Statistical analysis

Statistical significance between two groups was calculated using two-tailed student’s t-test. For groups of more than two, statistical significance was evaluated using ANOVA and then pairwise comparison using Tukey’s test was performed. Spearman correlation with clinical parameters was calculated using GraphPad Prism 6. For adjusting multiple testing, we employed Bonferroni correction. Logistic regression was performed to predict sample status (control vs. case) based on NAS or fibrosis scores. First, univariate logistic regression models were fitted for fatty acids and clinical variables. For each logistic regression model, the area under the receiver operating characteristic (ROC) curve (AUC) was calculated. Multivariate regression models were then built based on significant results from the univariate analysis. In order to compare models, a likelihood ratio test was conducted. For ROC analysis, leave-one-out cross validation prediction error was computed. All statistical analyses were performed using R software (http://www.r-project.org/).

## Results

### Circulating fatty acids in NAFLD patients according to NAFLD activity scores

**Fig 1:**
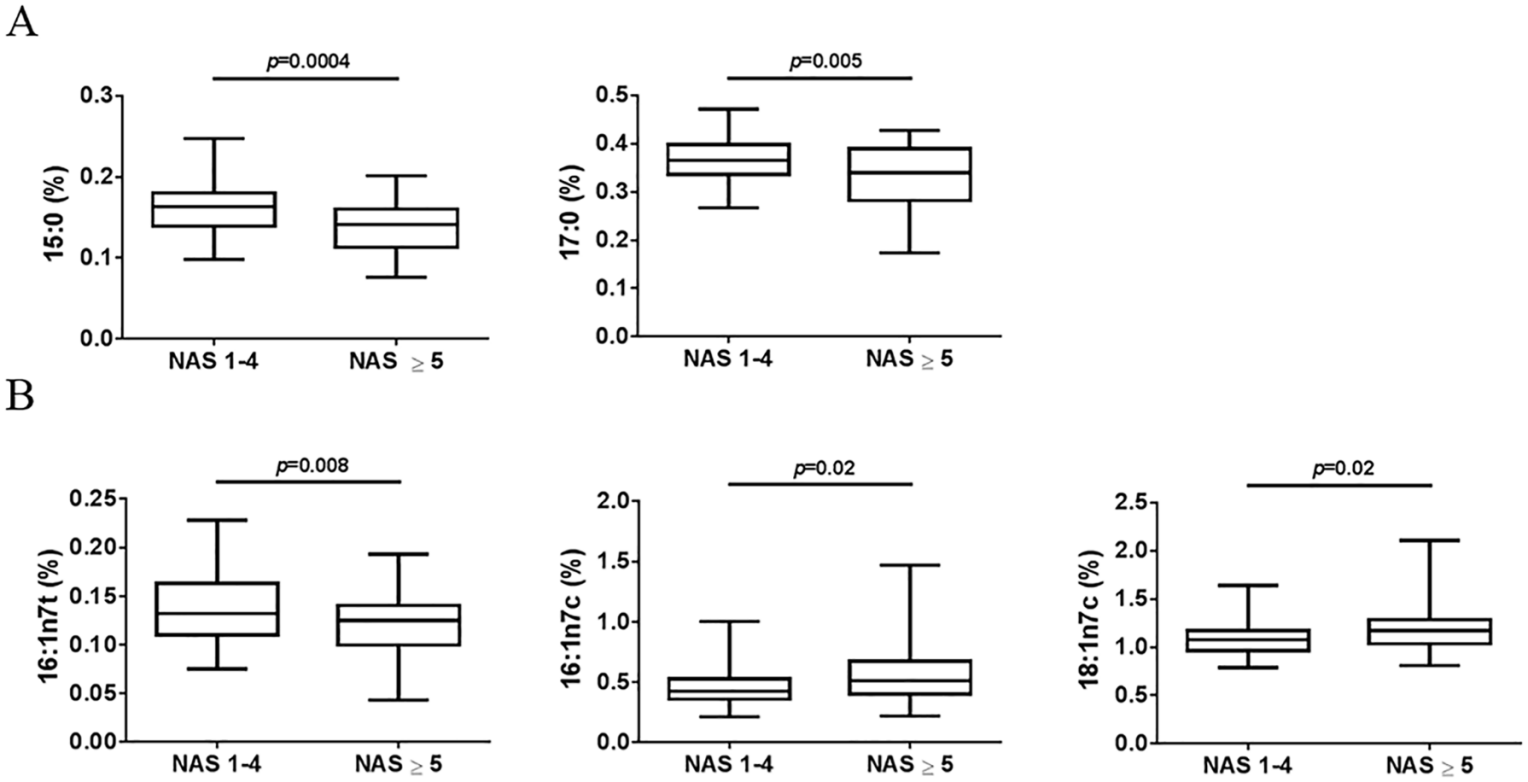
Fatty acids that showed significantly different levels in serum between patients with NAFLD activity scores (NAS) 1–4 (n = 68) and patients with NAS ≥5 (n = 38). (A) Saturated fatty acids (SFAs), pentadecanoic (15:0) and heptadecanoic (17:0) acid, (B) monounsaturated fatty acids (MUFAs), palmitelaidic (16:1n7t), palmitoleic (16:1n7c) and vaccenic (18:1n7c) acid. Fatty acids were measured as percentage of the phospholipid fraction. P-value was calculated using two-tailed student’s T-test.

To determine whether the composition of circulating fatty acids changes with disease severity in patients with NAFLD and could have utility in the diagnosis of NASH, we measured 46 fatty acids in sera collected from 106 patients with NAFLD. Based on the NAFLD activity score (NAS), we divided the NAFLD patients into two groups: patients with NAS 1–4 (n = 68) and patients with NAS ≥5 (n = 38). Significant differences were observed between the two groups in serum fatty acid compositions for odd chain saturated fatty acids (SFAs) and monounsaturated fatty acids (MUFAs). The odd chain SFAs, pentadecanoic acid (15:0) and heptadecanoic acid (17:0, margaric), were significantly lower in patients with NAS ≥5 compared to patients with NAS 1–4 (p = 0.0004 and p = 0.005, respectively) (Fig 1A). The MUFAs, palmitelaidic acid (16:1n7t), palmitoleic acid (16:1n7c) and vaccenic acid (18:1n7c), were also detected at different levels between patients with NAS 1–4 or NAS ≥5. Compared to patients with NAS 1–4, 16:1n7t levels were lower (p = 0.008) while levels of 16:1n7c and of 18:1n7c were higher in patients with NAS ≥5 (p = 0.02) (Fig 1B).

### Circulating fatty acids in NAFLD patients according to fibrosis severity

**Fig 2:**
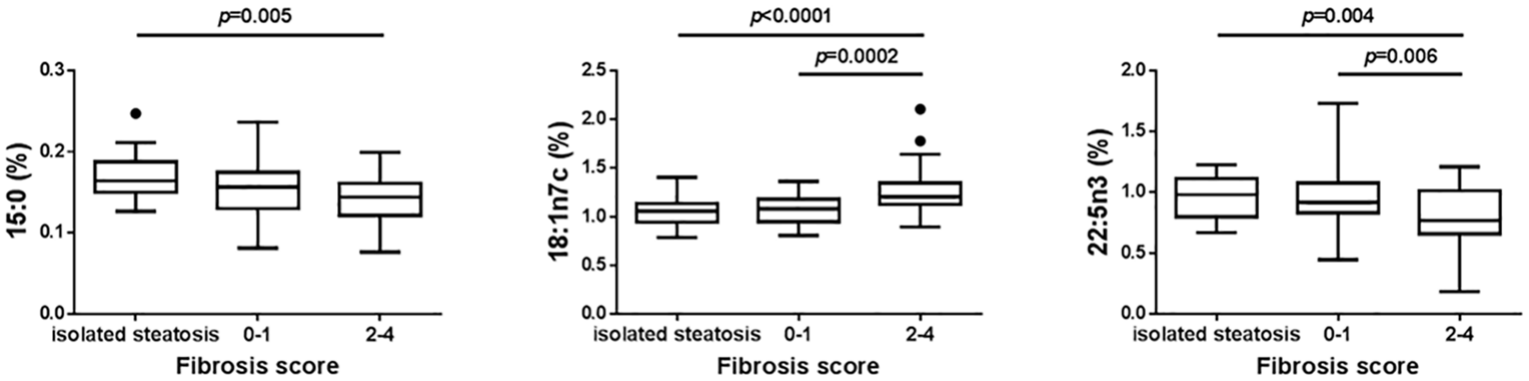
Pentadecanoic acid (15:0), vaccenic acid (18:1n7c) and docosapentaenoic acid (22:5n3) serum levels in patients. Isolated steatosis (n = 27), NASH patients with fibrosis stage 0–1 (n = 45) and NASH patients with fibrosis stage 2–4 (n = 34). Fatty acids were measured as percentage of the phospholipid fraction. Statistical significance was done using ANOVA test and then pairwise comparison using Tukey’s test was performed.

We also looked at fatty acid level differences when grouping the 106 NAFLD patients into patients with isolated steatosis (n = 27), patients with no or mild fibrosis (stages 0–1) (n = 45) and patients with intermediate to severe fibrosis (stages 2–4) (n = 34). Of the 46 fatty acids measured, this analysis identified again 15:0 and 18:1n7c and an additional fatty acid, docosapentaenoic acid (22:5n3). For these three fatty acids, the patient subgroups were significantly different from each other (p = 0.005, p<0.0001 and p = 0.004 for 15:0, 18:1n7c and 22:5n3, respectively) (Fig 2). Furthermore, levels of 18:1n7c were significantly higher in patients with fibrosis scores 2–4 compared to patients with fibrosis scores 0–1 (p = 0.0002) while levels of 22:5n3 were significantly lower in patients with fibrosis scores 2–4 compared to patients with fibrosis scores 0–1 (p = 0.006) (Fig 2).

### Circulating fatty acids correlation with parameters of NASH

**Fig 3:**
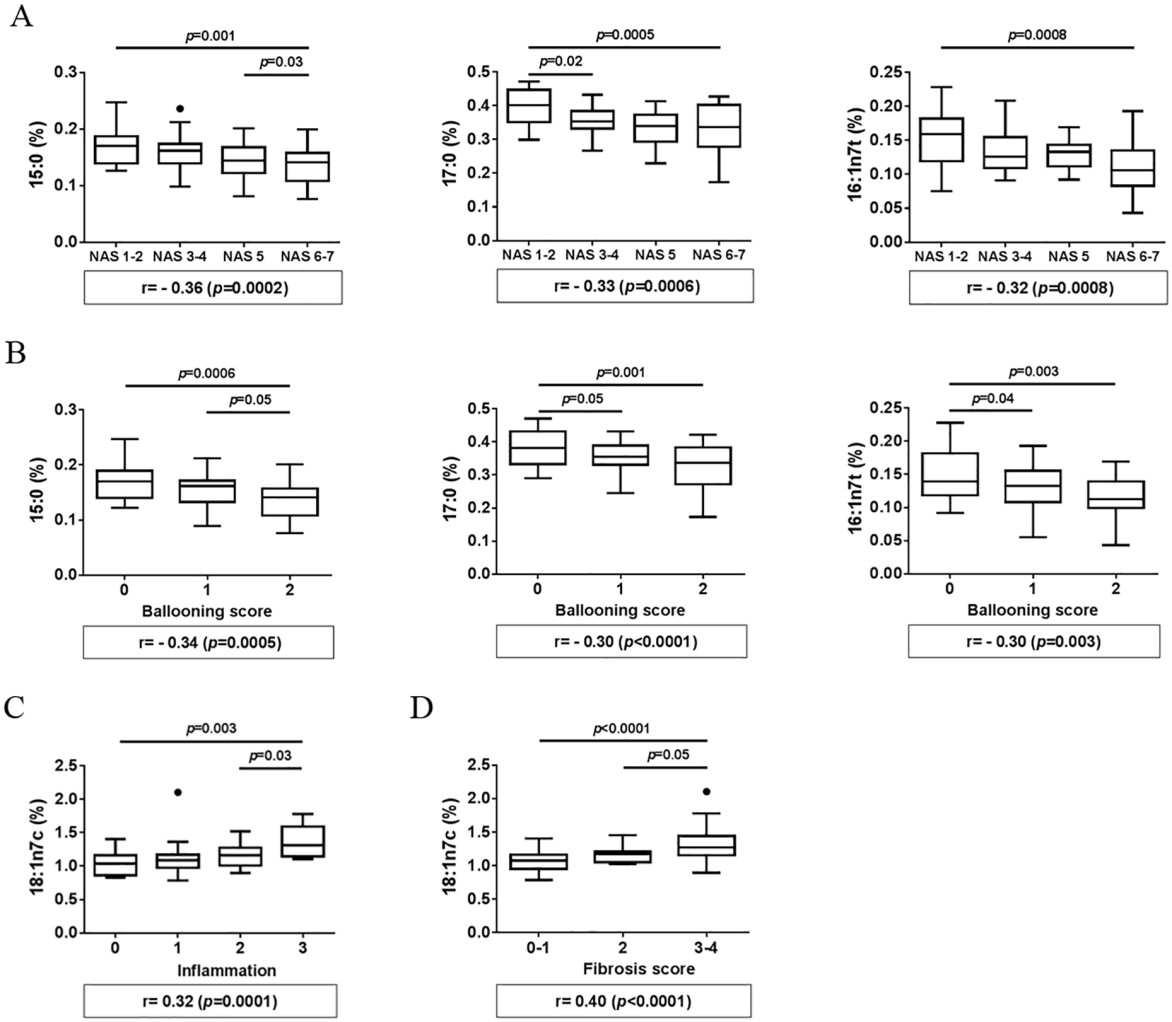
Correlation of fatty acids with NASH parameters. (A) Correlation of pentadecanoic (15:0), heptadecanoic (17:0) acid and palmitelaidic (16:1n7t) with NAFLD activity scores (NAS), (B) correlation of pentadecanoic (15:0), heptadecanoic (17:0) acid and palmitelaidic (16:1n7t) with hepatocyte ballooning scores, (C) correlation of vaccenic acid (18:1n7c) with liver inflammation scores, and (D) correlation of vaccenic acid (18:1n7c) with fibrosis scores. Fatty acid levels are presented as a percentage of the phospholipid fraction. Statistical significance was done using ANOVA test and then pairwise comparison using Tukey’s test was performed. Spearman correlation was calculated using GraphPad Prism 6 and Bonferroni correction was used to select significant correlations.

To further determine the relation between the 6 fatty acids identified (15:0, 17:0, 16:1n7t, 16:1n7c, 18:1n7c and 22:5n3) and NASH, we performed correlation analysis between these 6 fatty acids and parameters of NASH, including NAS, hepatocyte ballooning, inflammation, steatosis and fibrosis scores. A negative correlation with NAS scores was observed for 15:0, 17:0 and 16:1n7t (r = -0.36, p = 0.0002; r = -0.33, p = 0.0006 and r = -0.32, p = 0.0008, respectively). When patients were divided into four subgroups based on NAS (1–2, 3–4, 5 and 6–7), levels of 15:0, 17:0 and 16:1n7t progressively and significantly decreased as NAS increased in subgroups (p = 0.001, p = 0.0005 and p = 0.0008, respectively) (Fig 3A). Furthermore, levels of 15:0 were significantly lower in patients with NAS 6–7 compared to patients with NAS 5 (p = 0.03) and levels of 17:0 were significantly lower in patients with NAS 3–4 compared to patients with NAS 1–2 (p = 0.02) (Fig 3A). Interestingly, levels of these same fatty acids (15:0, 17:0 and 16:1n7t) negatively correlated with hepatocyte ballooning scores (r = -0.34, p = 0.0005; r = -0.30, p = 0.003 and r = -0.30, p = 0.003, respectively). Levels of 15:0, 17:0 and 16:1n7t were significantly different among the three liver ballooning score subgroups (p = 0.0006, p = 0.001 and p = 0.003, respectively) (Fig 3B). Furthermore, levels of 17:0 and 16:1n7t were significantly lower in patients with ballooning score 1 compared to patients with ballooning score 0 (p = 0.05 and p = 0.04, respectively) and levels of 15:0 were significantly lower in patients with ballooning score 2 compared to score 1 (p = 0.05) (Fig 3B). While none of the 6 fatty acids correlated with steatosis, levels of 18:1n7c positively correlated with inflammation (r = 0.32, p = 0.001) and had the ability to discriminate between patient subgroups separated based on inflammation scores (p = 0.003) (Fig 3C). In particular, 18:1n7c level was significantly higher in patients with inflammation score 3 compared to those with score 2 (p = 0.03). Levels of 18:1n7c also strongly correlated with fibrosis (r = 0.40, p<0.0001) (Fig 3D). When patients were divided into three groups based on fibrosis scores (mild: 0–1, intermediate: 2 and advanced: 3–4), levels of 18:1n7c showed significant difference among the three groups (p<0.0001) (Fig 3D). Furthermore, 18:1n7c levels were significantly higher in patients with advanced fibrosis (3–4) compared to patients with intermediate fibrosis (2) (p = 0.05) (Fig 3D).

### Circulating fatty acids correlation with parameters of liver injury and metabolic syndrome

**Fig 4:**
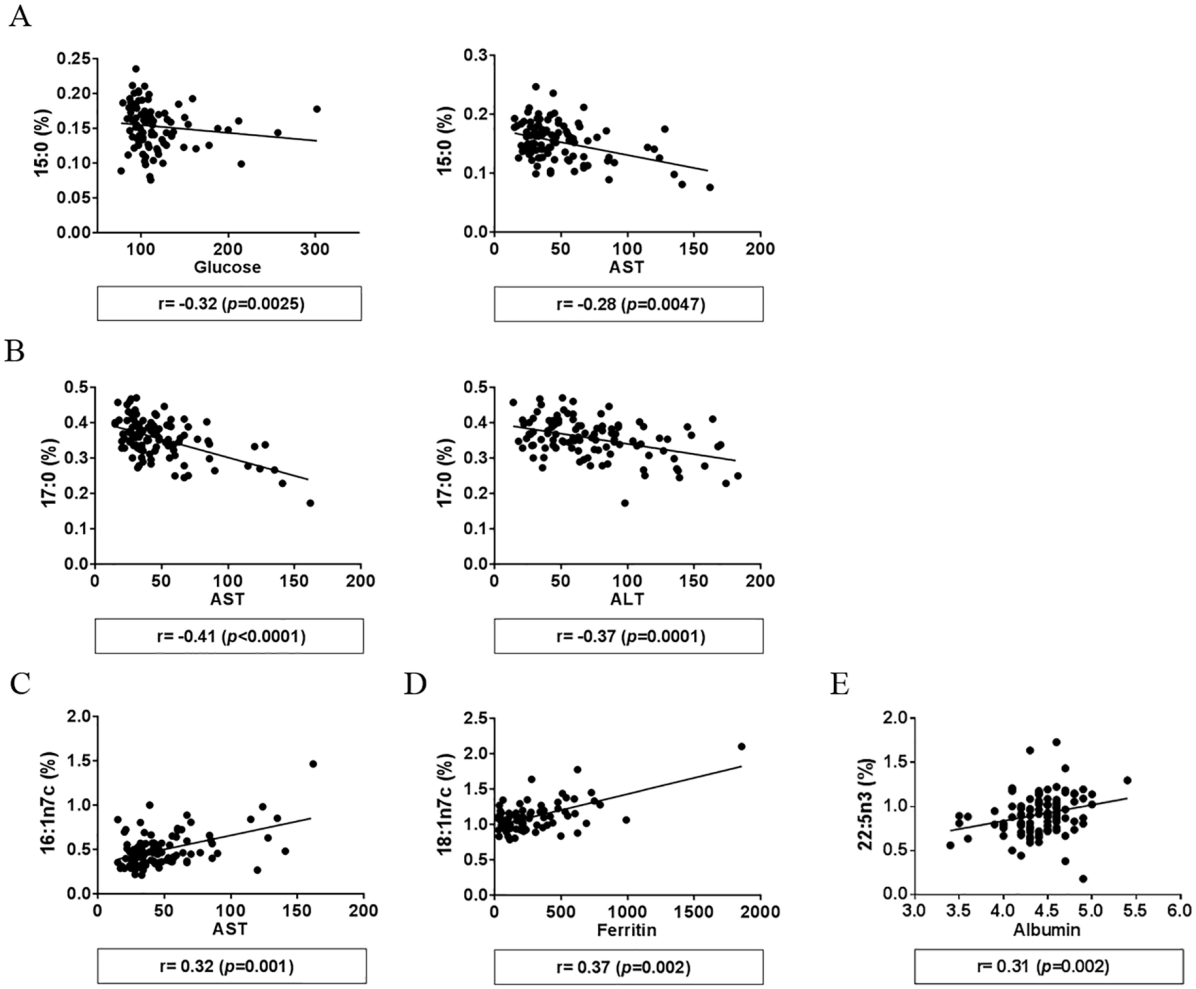
Correlation of fatty acid levels with parameters of liver injury and metabolic syndrome. (A) Correlation of pentadecanoic (15:0) with fasting glucose levels and aspartate transaminase (AST), (B) correlation of heptadecanoic (17:0) with AST and alanine transaminase (ALT) levels, (C) correlation of palmitoleic acid (16:1n7c) with AST levels, (D) correlation of vaccenic acid (18:1n7c) with ferritin levels, and (E) correlation of docosapentaenoic acid (22:5n3) with albumin levels. Spearman correlation was calculated using GraphPad Prism 6 and Bonferroni correction was used to select the significant correlations.

To extend the observed correlations between fatty acids and NASH parameters, we performed additional analysis relative to biochemical features of liver injury and metabolic syndrome. Levels of 15:0 negatively correlated with fasting glucose levels (r = -0.30, p = 0.0025) and aspartate transaminase (AST) (r = -0.28, *p* = 0.0047) (Fig 4A) while levels of 17:0 negatively correlated with AST and alanine aminotransferase (ALT) (r = -0.41, p<0.0001 and r = -0.37, p = 0.0001, respectively) (Fig 4B). Levels of 16:1n7c showed a positive correlation with AST (r = 0.32, p = 0.001) (Fig 4C). Additional correlations were observed between 18:1n7c and ferritin levels (r = 0.32, p = 0.002) (Fig 4D) and between 22:5n3 and albumin levels (r = 0.31, p = 0.002) (Fig 4E). None of the six fatty acids correlated with BMI.

### Receiver operating characteristic (ROC) for biomarker performance

**Fig 5:**
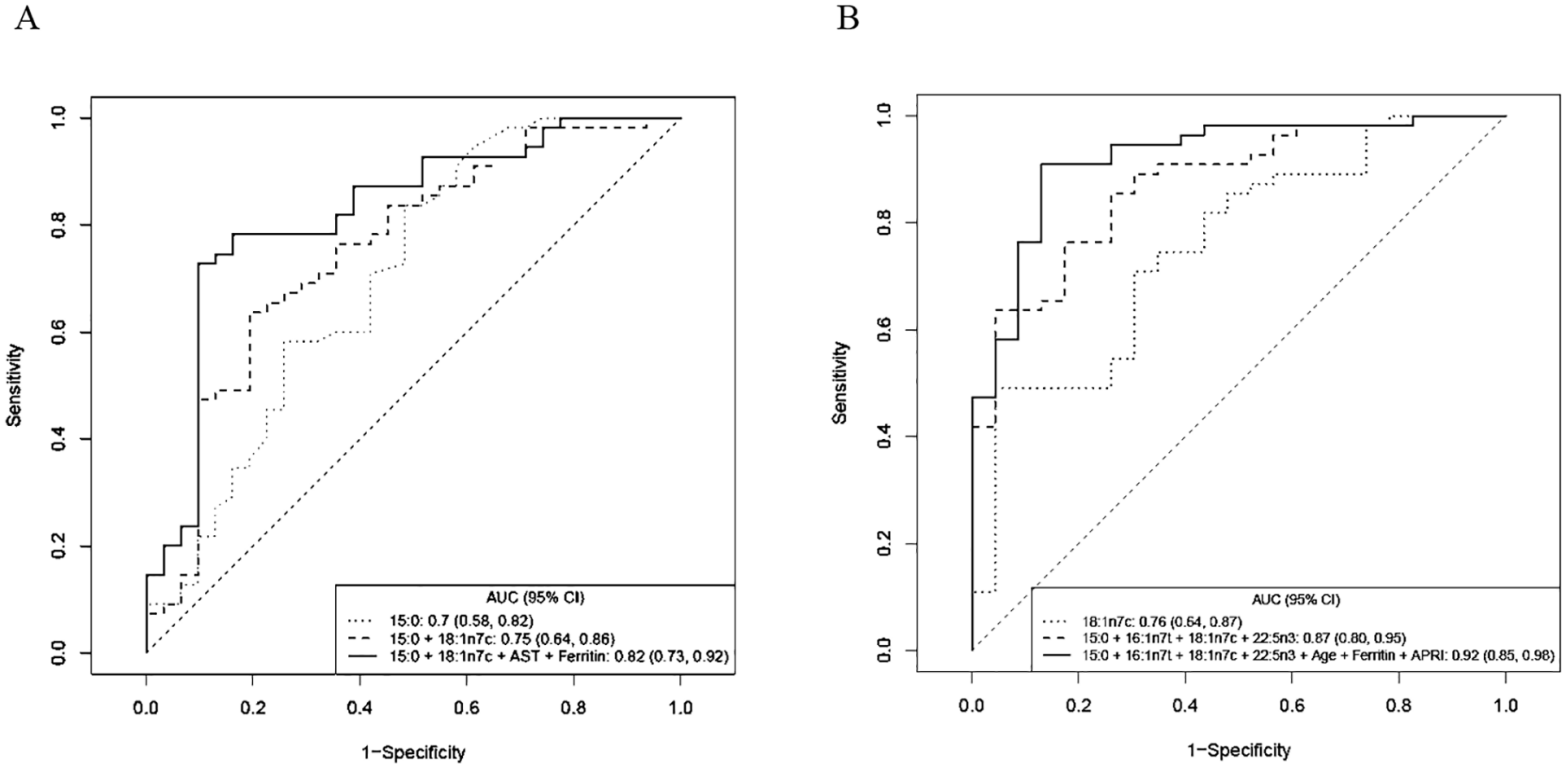
Receiver operating characteristic (ROC) analysis. (A) based on NAFLD activity scores (NAS)–patients with NAS 1–4 versus patients with NAS ≥5. (B) Based on fibrosis scores—patients with fibrosis 0–1 versus patients with fibrosis 2–4.

To further evaluate the performance of the selected fatty acids to assess disease severity in NAFLD patients, we used receiver operating characteristic (ROC) curves. When used individually, 15:0 had the best performance for predicting NAS ≥5 with an area under the receiver-operator curve (AUC) value of 0.70 (95% CI: 0.58–0.82, p = 0.0002). The performance improved when 15:0 was combined with 18:1n7c [AUC = 0.75 (95% CI: 0.64–0.86, p<0.0001). Adding AST and ferritin to the two fatty acid panel further improved performance with AUC = 0.82 (95% CI: 0.73–0.92, p<0.0001) (Fig 5A). This combination had a sensitivity of 73% at 90% specificity and the prediction error rate of the model was 18%.

We also performed ROC curve analyses to evaluate the performance of fatty acids in discriminating NAFLD patients with fibrosis stage 0–1 and NAFLD patients with fibrosis stage 2–4. In this analysis, 18:1n7c had the best performance with an AUC of 0.76 (95% CI: 0.64–0.87, p<0.0001). Combining 18:1n7c with 15:0, 16:1n7t and 22:5n3 improved the AUC to 0.87 (95% CI: 0.80–0.95, p<0.0001). Performance was further improved when age, ferritin and AST-to-Platelet ratio (APRI), were added to the 4 fatty acid panel (AUC = 0.92–95% CI: 0.85–0.98, p<0.0001) (Fig 5B). This combination had a sensitivity of 82% at 90% specificity and the prediction error rate of the model was 13%.

### In vivo evaluation of 15:0 and 18:1n7c treatment

**Fig 6:**
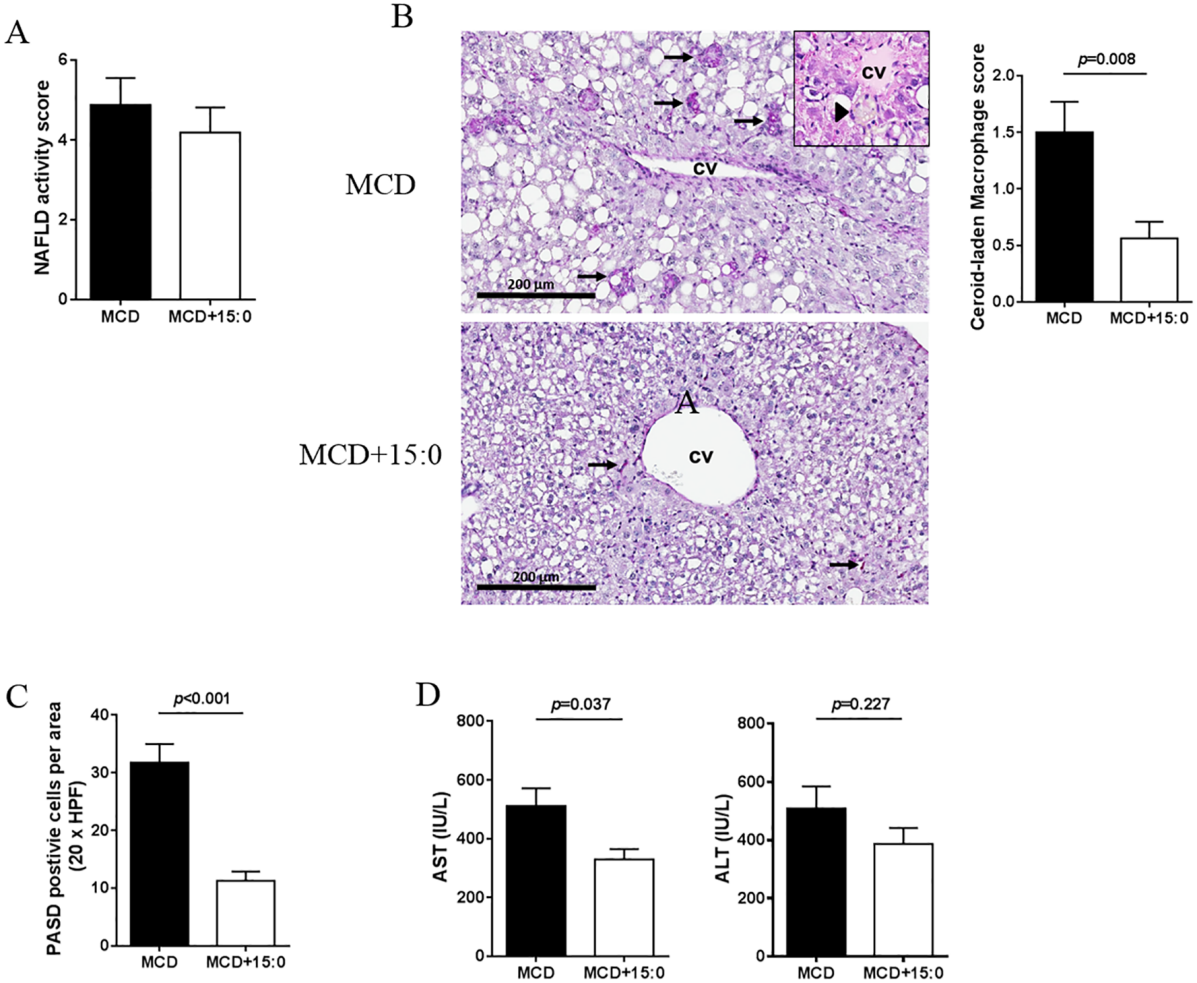
Effects of 15:0-supplemented MCD diet *in vivo*. (A) NAS scores from H&E stained slides. (B) Histologic evaluation by H&E and (C) Periodic acid Schiff with diastase (PAS-D) staining in liver from mice treated with MCD or with MCD+15:0. (D) AST and ALT levels in mice treated with MCD or with MCD+15:0.

To investigate whether the identified fatty acids directly contribute to NASH pathogenesis, we first evaluated whether supplementing the diet with 15:0 or with 18:1n7c, in mice treated with methionine and choline deficient (MCD) diet, a well-characterized dietary NASH model, would result in inhibition or acceleration of the NASH phenotype. No significant difference in NAFLD activity score was observed in mice treated with MCD+15:0 or with MCD (Fig 6A). However, significant differences were observed in the presence of ceroid-laden macrophages. Mice treated with MCD presented with frequent ceroid-laden macrophages which were most prominent near the central veins (Fig 6B). Large aggregates of these cells were visible in the H&E-stained sections (Fig 6B). Liver of mice treated with MCD supplemented with 5% 15:0 (MCD+15:0) had significantly fewer ceroid-laden macrophages and these macrophages were smaller in size (Fig 6B). In addition, the large clusters and aggregates of macrophages observed in the MCD-treated mice were not observed. Overall, the average ceroid-laden macrophage score was reduced from 1.5 ± 0.27 to. 0.56 ± 0.15 (p = 0.008) upon 15:0 treatment (Fig 6B). The reduction of ceroid-laden macrophages in mice treated with MCD+15:0 compared to MCD was further confirmed by periodic acid–Schiff with diastase digestion (PASD) staining (31.76 ± 3.24 vs. 11.29 ± 1.59, p<0.001) (Fig 6C). AST (but not ALT) levels were also significantly decreased in mice treated with MCD+15:0 compared to mice treated with MCD (511.2 ± 60.46 vs. 329.3 ± 34.99, p = 0.037) (Fig 6D). In addition, liver weight (0.67 ± 0.07 vs 0.57 ± 0.05, p = 0.009) and liver-to-body weight ratio (0.044 ± 0.001 vs 0.037 ± 0.001, p = 0.009) were significantly higher in mice subjected to the 15:0 supplement than in MCD fed mice, further confirming that the 15:0 supplement significantly reduced liver injury resulting from MCD diet (Table 2). Altogether, these results are in agreement with our observation that circulating 15:0 negatively correlated with liver injury, AST levels but not ALT levels, in NAFLD patients and suggest that 15:0 is a promising therapeutic target. We then evaluated the role of 18:1n7c on NASH pathogenesis. No changes were observed in histology and physiological parameters, nor in the expression of the inflammation-associated genes, TNF-α and IL-6 as shown in supporting information (S1 Fig and S1 Table) after treatment of MCD+181n7c. Serum AST and ALT levels were also unchanged upon 18:1n7c treatment (data not shown).

**Table 2:** Comparison of physiological parameters with 15:0 treatments *in vivo*.

	MCD (n = 8)	MCD+15:0 (n = 8)	*p*-value
**Liver weight (g)**	0.57 ± 0.05	0.67 ± 0.07	0.009
**Liver/Body ratio**	0.037 ± 0.00	0.044 ± 0.00	0.009

MCD, methionine and choline deficient diet; MCD+15:0, methionine and choline deficient diet plus 5% 15:0 fatty acid diets. Data were presented as the mean ± SD.

## Discussion

A key challenge in the diagnosis and therapy of NAFLD is distinguishing NASH from simple steatosis and reliably assessing disease stage. Nonalcoholic steatosis and NASH are stages in the spectrum of NAFLD. Scoring systems based on clinical and laboratory variables or imaging can assist in predicting NASH but none of them yet can replace liver biopsy.[17, 18] Non-invasive diagnostic tools for the differentiation of fatty liver from NASH as well as for the determination of presence and extent of fibrosis are urgently needed. In the current study, we performed a comprehensive evaluation of circulating fatty acids in well phenotyped patients with NAFLD and correlated findings with liver histology and biochemical parameters of liver injury and metabolic syndrome. We identified a panel of six circulating fatty acids that could have utility in detecting NASH among patients with NAFLD. These include the odd chain saturated fatty acids 15:0 (pentadecanoic acid) and 17:0 (heptadecanoic/margaric acid), the monounsaturated fatty acids 16:1n7t (palmitelaidic acid), 16:1n7c (palmitoleic acid) and 18:1n7c (vaccenic acid) and the n3-polyunsaturated fatty acid 22:5n3 (docosapentaenoic acid, DPA). Levels of 15:0, 17:0 and 16:1n7t negatively correlated with NAS scores and ballooning scores, while levels of 18:1n7c strongly correlated with fibrosis scores and significantly discriminated patients with intermediate or advanced fibrosis from patients with mild fibrosis. The panel composed of 15:0, 16:1n7t, 18:1n7c, 22:5n3, age, ferritin and AST-to-Platelet Ratio Index (APRI) best detected NAFLD patients with intermediate or advanced fibrosis, with an area under ROC curve (AUROC) of 0.92 and a 82% sensitivity at 90% specificity. Limitations of the study include the relatively small overall sample size, the lack of significant representation of Asian and African American ethnicities and the lack of an independent review of the liver histology interpretation and scoring. Limitations of the study also include the narrow analysis of the correlations between fatty acids and individual histological features or composite NAS score. Additional studies are needed to further evaluate the performance of the identified fatty acid-based model in the diagnosis of NASH in patients with NAFLD.

Most biomarker studies performed to date to evaluate NAFLD histology were not performed on fatty acids.[19–26] Several studies used lipidomics or metabolomics approaches, including a recent report proposing a panel of 3 metabolites and 2 lipids combined to PNPLA3 genetic variant for the detection of NASH [AUROC = 0.86].[26] However, some studies have measured fatty acids in plasma or in liver in the context of NAFLD. In these studies, the panel of fatty acids measured was smaller than in our study that interrogated 46 different fatty acids. In liver, decreased levels of 20:4n6 (arachidonic acid), 20:5n3 (eicosapentanoic acid, EPA) and 22:6n3 (docosahexanoic acid, DHA) were observed in triacylglycerol lipids fraction.[27] Another study measured fatty acid composition in liver and identified increased 16:1n7c in NASH.[28] Only one study was performed in plasma and reported higher levels of circulating 16:1n7 (palmitoleic acid) and desaturase activity in NAFLD compared to normal controls.[29] In our study, we detected high levels of circulating 16:1n7c and of 18:1n7c, the elongated product of 16:1n7c in NAFLD patients with NAS ≥5.

While prior studies described decreased levels of 20:5n3 and 22:6n3 in NAFLD liver.[26] we identified decreased circulating levels of another n3 PUFA, 22:5n3, in NASH patients with intermediate to severe fibrosis. Recent studies testing the effects of n3 PUFA in patients with NAFLD are showing promise.[30, 31] More specifically, n3 PUFA supplementation may decrease liver fat.[32, 33] However, no improvement of fibrosis or other metabolic effect has been observed.[33, 34] To date, all studies have used 20:5n3 (EPA) and 22:6n3 (DHA) while our study identified 22:5n3 (DPA) suggesting that further research is needed to establish the optimal composition of n3 therapy. Whether there is benefit on the different component features of NAFLD (hepatic fat, inflammation, and fibrosis) should also be further tested.

We also identified novel fatty acid changes in NASH, such as lower levels of the odd chain SFAs, 15:0 and 17:0, and of the *trans*-MUFA, 16:1n7t. The odd chain saturated fatty acids 15:0 and 17:0 were first thought to mainly derive from consumption of dairy fat and originate from biosynthesis in rumen microbiome.[35, 36] 16:1n7t has also been associated with dairy fat consumption. The ratio of 15:0 to 17:0 is approximately 2:1 in ruminant milk fat, while it is approximately 1:2 in human tissues and biofluids, contradicting the hypothesis that the source of these fatty acids is exclusively diet.[35] The ratio 15:0 to 17:0 in our analysis was also 1:2. Recent studies suggested that bio-synthesis of odd chain fatty acids can occur through alpha-oxidation in adipose tissue. Both 15:0 and 17:0 have been shown to have a positive association with health and in particular with risk for type 2 diabetes and cardiovascular diseases.[35, 37–39]

An important novelty in our study is the extensive correlation analysis performed between levels of the six fatty acids identified and parameters of NASH, liver injury and metabolic syndrome. Of great interest is the observation that 15:0, 17:0 and 16:1n7t negatively correlated with hepatocyte ballooning. Cellular ballooning is a histologic hallmark of NASH and correlates with disease progression. There are complex correlations between ballooning, Mallory-Denk bodies and apoptosis and whether apoptosis may promote hepatocellular ballooning, or vice versa, is a subject of study.[40] Whether these fatty acids modulate apoptosis and whether diet supplementation with these fatty acids, reduce hepatocyte ballooning in mouse models of NASH in under investigation. The strongest correlation was observed between 18:1n7c levels and fibrosis severity (positive correlation) and between 17:0 levels and liver function enzymes AST and ALT (negative correlation). Interestingly, 18:1n7c also strongly correlated with ferritin levels. Serum ferritin levels are commonly elevated in patients with NAFLD because of systemic inflammation and increased iron stores. Elevated serum ferritin is independently associated with higher NAS, even among patients without hepatic iron deposition and is an independent predictor of histologic severity and advanced fibrosis in NAFLD patients.[41] The NAFLD score, calculated from the levels of ferritin, fasting insulin, and type IV collagen 7S, has been proposed for the diagnosis of NASH. It has to be noted that none of the six fatty acids correlated with BMI.

Because of the remarkable correlations we observed between the fatty acids 15:0 and 18:1n7c and hallmarks of NASH, hepatocytic ballooning and fibrosis scores in particular, and because of the contribution of these two fatty acids to the NASH diagnosis panel we identified, we investigated whether 15:0 and 18:1n7c levels could play a role in the pathogenesis of NASH. To that end, we used the dietary MCD NASH mouse model that has been widely used to study modifiers of NASH phenotype and that has been validated as a highly relevant model to study the pathological mechanisms that cause human NAFLD to progress to advanced NASH.[12, 42–44] We demonstrated that 15:0 is a regulator of liver injury as measured by AST levels and that a 15:0 supplement diet reduces the number of ceroid-laden macrophages. In contrast, treatment of mice with 18:1n7c supplement diet showed no significant change in all NASH parameters.

In summary, the comprehensive analysis of fatty acid composition through the different stages of NAFLD and their correlation to histological hallmarks of NASH described in this study, identified novel biomarkers of elevated NAS and of advanced fibrosis. It also provided insights into mechanisms of disease progression. A panel including fatty acid biomarkers and clinical parameters was sufficient to discriminate between histologically defined categories of NAFLD. The most performant panel in discriminating NAFLD patients with mild fibrosis and NAFLD patients with intermediate or advanced fibrosis consisted of a panel of the four fatty acids, 15:0, 16:1n7t, 18:1n7c and 22:5n3, and age, ferritin and APRI, with a performance of 82% sensitivity at 90% specificity. The identified panels in our study outperform previously reported marker panels or scoring systems, recently evaluated in a systematic review and meta-analysis.[45] The identified panels should be further evaluated in prospective cohorts for its utility in distinguishing histological severity in patients with NAFLD. In addition, the fatty acid 15:0 was identified as a modulator of liver injury parameters in preclinical models of NASH, while 18:1n7c had no direct effect on NASH pathology. A diet supplemented with 15:0 could improve liver inflammation and injury in NASH patients and should be further investigated as a therapeutic approach to prevent progression to NASH in patients with NAFLD.

## Supporting information

S1 Table Comparison of physiological parameters with 18:1n7c treatments *in vivo*. (DOCX)

S1 Fig Effects of 18:1n7c-supplemented MCD diet in vivo. (A) NAS, lobular inflammation and ballooning scored blindly by a pathologist. (B) Hepatic mRNA expression of inflammatory markers, IL-6 and TNF-ɑ measured by qRT-PCR in mice fed the MCD or MCD+18:1n7c.
(TIF)
