# Supplementary material for: Fatty acids in non-alcoholic steatohepatitis: Focus on pentadecanoic acid
Source: PLoS One. 2017 Dec 15;12(12):e0189965. doi: 10.1371/journal.pone.0189965 (PMC5731750; doi:10.1371/journal.pone.0189965)
Supplement: S1 Table — (DOCX) [file pone.0189965.s001.docx]

**S1 Table. Comparison of physiological parameters with 18:1n7c treatments *in vivo***

|  | **MCD (n=8)** | **MCD+18:1n7c (n=8)** | ***p*-value** |
| --- | --- | --- | --- |
| **Liver weight (g)** | 0.60 ± 0.05 | 0.61 ± 0.10 | NS |
| **Liver/Body ratio** | 0.038 ± 0.002 | 0.038 ± 0.005 | NS |
| MCD, methionine and choline deficient diet; MCD+18:1n7c, methionine and choline deficient diet plus 5% 18:1n7c fatty acid diet. Data were presented as the mean ± SD. NS: Not significant | | | |
