# Supplementary figures and images for: Fatty acids in non-alcoholic steatohepatitis: Focus on pentadecanoic acid
Source: PLoS One. 2017 Dec 15;12(12):e0189965. doi: 10.1371/journal.pone.0189965 (PMC5731750; doi:10.1371/journal.pone.0189965)

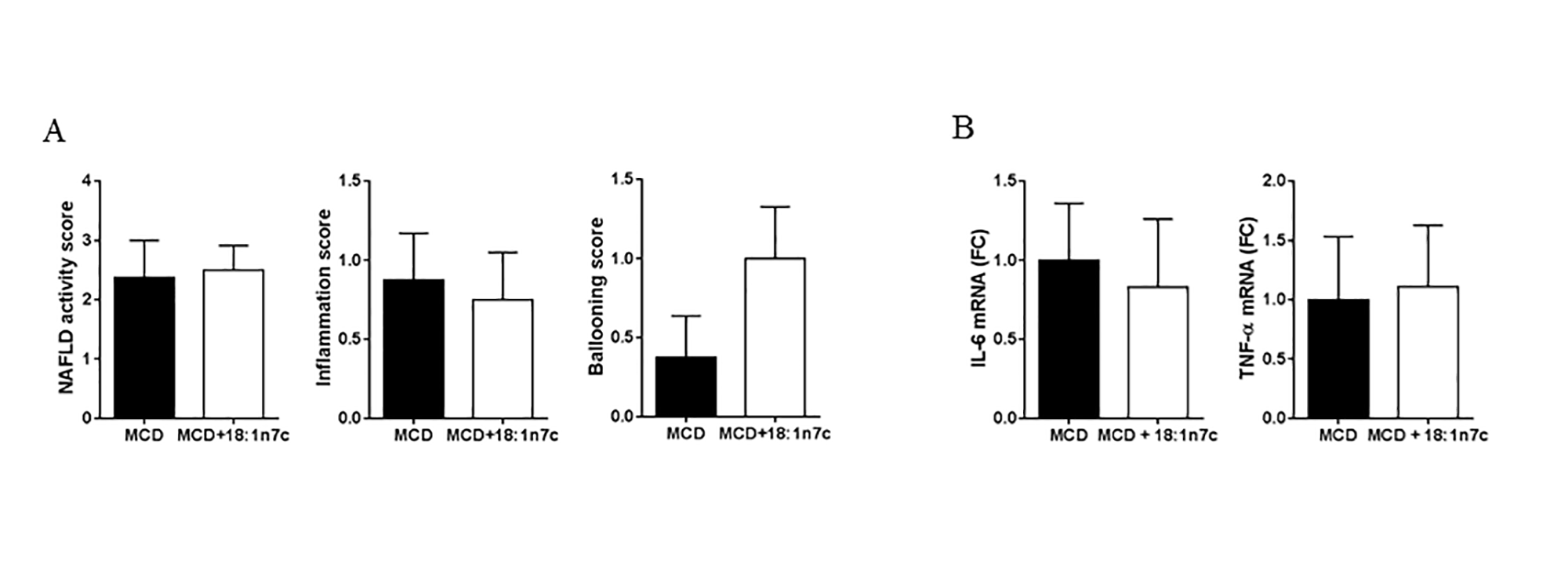

Supplement: S1 Fig — (A) NAS, lobular inflammation and ballooning scored blindly by a pathologist. (B) Hepatic mRNA expression of inflammatory markers, IL-6 and TNF-ɑ measured by qRT-PCR in mice fed the MCD or MCD+18:1n7c. (TIF) [file pone.0189965.s002.TIF]
